# Editorial: Constructing new motifs in hematology

**DOI:** 10.3389/fmed.2023.1220022

**Published:** 2023-06-06

**Authors:** Erden Atilla

**Affiliations:** Fred Hutchinson Cancer Research Center, Clinical Research Division, Seattle, WA, United States

**Keywords:** anemia, Myelodysplastic Syndrome, lymphoma, granulocyte infusion, coagulation

The word “hematology” was referred to first in 1748 with the publication of Thomas Schwenke's book, Haematologia, a “treatise as complete as it could then be, upon the blood considered in its state of health and of disease” (1). From then on, tremendous efforts were put forth to evolve hematology with the influences of the application of novel diagnostic technologies, genetic revolution, and molecular mechanisms to lighten the challenges in the pathogenesis of diseases. In the current Research Topic, we aimed to update the community on the new era in hematology, with state-of-the-art knowledge in all fields of hematology including recent diagnostic technologies, novel treatment approaches of malign/benign hematological diseases, and unsolved issues.

Anemia, the reduction in hemoglobin (Hb) or hematocrit (HCT) or red blood cells, is the most common blood disorder. Research is being undertaken for methods of detection, genetic and molecular aspects in pathophysiology as well as treatment approaches. Voxelotor is an HBs polymerization inhibitor approved by the FDA that has demonstrated an improved Hb level, and reduced hemolysis, with an improved rate of vaso-occlusive crises (2). Alkindi et al. defined the abnormal HbD variant on high-performance liquid chromatography (HPLC) in patients under Voxelotor, which will provide data on patient compliance. β-thalassemia is another inherited hemoglobin disorder mainly affecting the Mediterranean area, North and Central Africa, Southeast Asia, and the Middle East (3). Sanchez-Villalobos et al. reviewed the latest advances in the pathophysiology of β-thalassemia by categorizing it into three categories: correction of the globin chain imbalance, reverse ineffective erythropoiesis, and improving iron overload. As potential targets for future use, inflammasomes and HSP70 nuclei regulation were demonstrated (4, 5).

Myelodysplastic Syndrome (MDS) is a heterogeneous group of diseases in which ineffective hematopoiesis and cytopenia are predominant. Toprak published a detailed review of Low-Risk MDS including an updated definition, classification, pathogenesis, clinical presentation, risk stratification, prognostic assessment, and treatment. The presence of somatic mutations has been demonstrated, with TP53, ASXL1, EZH2, ETH2, ETV6, and RUNX1 showing a poor clinical course, whereas SF3B1 leads to good clinical outcomes defined with next-generation sequencing (NGS) (6). Interestingly, Zou et al. reported a case of MDS with an abnormal karyotype- t(11;22)(q23;q11) and the presence of an MLL-SEPT5 fusion transcript that is uncommon in MDS and more likely presents in adult and pediatric leukemia (7). For differential diagnosis between MDS, aplastic anemia (AA), and megaloblastic anemia (MA), Zhao et al. demonstrated a rapid and efficient method by identifying the area of red blood cells in peripheral blood smears based on the image processing technology, which might be an alternative in low-resource settings.

In one of the manuscripts included in this Research Topic, Chang et al. described a novel phenotype of the Factor V Gene Mutation (Homozygote Met1736Val and Heterozygote Asp68His) in the Asian population. Although the risk of bleeding in moderate Factor V deficiency is low (8), one of the cases presented a massive postpartum hemorrhage, which should be considered for future cases. In the next article, He et al. conducted a retrospective study among 121 chronic active Epstein-Barr virus infection (CAEBV) disease that progressed to hemophagocytic lymphohistiocytosis (HLH), which is characterized by multi-organ dysfunction due to excessive immune activation (9). They explored the risk factors and generated a nomogram to predict the risk of progression by plasma EBV-DNA load, platelet count, elevated alanine aminotransferase, and ≥2 of 3 lineages of cytopenia.

Granulocyte transfusions (GCs) are a potential therapy for neutropenic patients with infections resistant to antibiotics and anti-fungal drugs (10). Murru et al. investigated unknown points in various manufacturing strategies of granulocyte transfusions by evaluating leukocyte composition, neutrophil viability, calcium mobilization, chemotaxis, phagocytosis, reactive oxygen species, cytokine production, and metabolites. G-CSF GCs contained more neutrophils than prednisone GCs. Prednisone GC neutrophils showed enhanced phagocytosis and G-CSF GC neutrophils exhibited decreased chemotaxis but increased IL-8 production. G-CSF neutrophils seemed to be more sensitive to storage. A review by Castagna et al. lightens up a controversial issue in the care of relapsed/refractory aggressive lymphomas (large B-cell lymphoma and MCL) by results obtained after allo-HSCT and CAR T cell therapies.

In summary, today, excessive data have been accumulated in malign and benign hematology. Novel diagnostic methods sculpt the concept of “personalized medicine” rather than the conventional approach.

## Author contributions

The author confirms being the sole contributor of this work and has approved it for publication.

## References

[1] Aird William. Discoveries in Hematology Before the 1950s. Were Made by Non-Hematologists (2021). Available online at: https://www.thebloodproject.com/copy-did-you-know-2 (accessed May 08, 2023).

[2] Vichinsky E Hoppe CC Ataga KI Ware RE Nduba V El-Beshlawy A. A phase 3 randomized trial of voxelotor in sickle cell disease. N Engl J Med. (2019) 381:509–19. 10.1056/NEJMoa1903212 31199090

[3] Kattamis A Forni GL Aydinok Y Viprakasit V. Changing patterns in the epidemiology of beta-thalassemia. Eur J Haematol. (2020) 105:692–703. 10.1111/ejh.13512 32886826 PMC7692954

[4] Schroder K Tschopp J. The inflammasomes. Cell. (2010) 140:821–32. 10.1016/j.cell.2010.01.040 20303873

[5] Guillem F Dussiot M Colin E Suriyun T Arlet JB Goudin N. XPO1 regulates erythroid differentiation and is a new target for the treatment of beta-thalassemia. Haematologica. (2020) 105:2240–9. 10.3324/haematol.2018.210054 33054049 PMC7556489

[6] Bejar R Papaemmanuil E Haferlach T Garcia-Manero G Maciejewski JP Sekeres MA. Somatic mutations in MDS patients are associated with clinical features and predict prognosis independent of the IPSS-R: analysis of combined datasets from the International Working Group for Prognosis in MDS-Molecular Committee. Blood. (2015) 126:907. 10.1182/blood.V126.23.907.907

[7] El Chaer F Keng M Ballen KK. MLL-rearranged acute lymphoblastic leukemia. Curr Hematol Malig Rep. (2020) 15:83–9. 10.1007/s11899-020-00582-5 32350732

[8] van Wijk R Nieuwenhuis K van den Berg M Huizinga EG van der Meijden BB Kraaijenhagen RJ. Five novel mutations in the gene for human blood coagulation factor V associated with type I factor V deficiency. Blood. (2001) 98:358–67. 10.1182/blood.V98.2.358 11435304

[9] Henter JI Horne A Aricó M Egeler RM Filipovich AH Imashuku S. HLH-2004: diagnostic and therapeutic guidelines for hemophagocytic lymphohistiocytosis. Pediatr Blood Cancer. (2007) 48:124–31. 10.1002/pbc.21039 16937360

[10] Cugno C Deola S Filippini P Stroncek DF Rutella S. Granulocyte transfusions in children 466 and adults with hematological malignancies: benefits and controversies. J Transl Med. (2015) 13:362. 10.1186/s12967-015-0724-5 26572736 PMC4647505

